# Complexes Formed by Hydrophobic Interaction between Ag-Nanospheres and Adsorbents for the Detection of Methyl Salicylate VOC

**DOI:** 10.3390/nano9111621

**Published:** 2019-11-15

**Authors:** Jinhyuk Park, J. Alex Thomasson, Sandun Fernando, Kyung-Min Lee, Timothy J. Herrman

**Affiliations:** 1 Department of Biological and Agricultural Engineering, Texas A&M University, College Station, TX 77843, USA; thomasson@tamu.edu (J.A.T.); sfernando@tamu.edu (S.F.); 2 Office of the Texas State Chemist, Texas A&M AgriLife Research, Texas A&M University System, College Station, TX 77841, USA; kml@otsc.tamu.edu (K.-M.L.); tjh@otsc.tamu.edu (T.J.H.)

**Keywords:** surface-enhanced Raman spectroscopy (SERS), Ag-nanosphere (AgNS), phase transfer, surfactant, electrostatic interaction, volatile organic compounds (VOCs)

## Abstract

Surface-enhanced Raman spectroscopy (SERS) has been widely investigated in many applications. However, only little work has been done on using SERS for the detection of volatile organic compounds (VOCs), primarily due to the challenges associated with fabricating SERS substrates with sufficient hotspots for signal enhancement and with the surface interfacially compatible for the VOCs. This study investigated the phase transfer of Ag-nanospheres (AgNSs) from the aqueous phase to the non-aqueous phase by electrostatic interaction induced by cationic surfactants, and the feasibility of the transferred AgNSs as SERS substrates for the determination of methyl salicylate VOC. Results indicated that one of three cationic surfactants, tetraoctylammonium bromide (TOAB) dissolved in organic solvent showed successful phase transfer of the AgNSs confirmed by several characterization analyses. The complex formed by hydrophobic interaction between the transferred AgNSs and Tenax-TA adsorbent polymer was able to be utilized as a SERS substrate, and the volatile of methyl salicylate could be easily determined from SERS measurements at 4 h static volatile collection. Therefore, the proposed new techniques can be effectively employed to areas where many VOCs relevant to food and agriculture need to be analyzed.

## 1. Introduction

Plant-emitted volatile organic compounds (VOCs) are important constituents that may indicate a plant’s physiological damage, potentially induced by abiotic or biotic stresses. Gas-chromatography/mass spectrometry (GC/MS) has been widely used to analyze VOCs due to its high sensitivity, but a great deal of time is required for the analysis, and it is not suitable for field application. Therefore, surface-enhanced Raman spectroscopy (SERS) using a metallic nanostructure for signal enhancement is considered a potential alternative in field applications due to its reasonable sensitivity, selectivity, and fast response [1]. To determine VOCs with SERS, key configuration requirements involve (1) using as many “hotspots” on nanoparticles as possible to intensify the weak Raman signal from the VOCs [2], and (2) making the surface properties of the nanoparticle favorable to the VOCs so they can be adsorbed on the surface due to enhanced interfacial compatibility [3].

There is greater need to develop 3D rather than 2D SERS substrates due to their stronger signal enhancement caused by the higher density of hotspots in 3D structures [4]. Most 3D substrates have been designed to make each nanoparticle aggregate unique, because signal enhancement at the gap between particles can be much stronger than at the surface of a single nanoparticle [5]. Research on fabricating nanoparticle aggregates as a SERS substrate has been done by several research groups.

An initial approach was to randomly deposit particle aggregates on a substrate. An Ag-nanoparticle (AgNP) aggregate was formed on copper foil by immersing the foil into the particle solution [6], and the AgNPs were also deposited on a glass substrate by a high-pressure sputtering technique [7]. However, there was a lack of regularity in the number of hotspots, resulting in an irreproducible and weak signal. Thus, it was necessary to develop a substrate with more uniform distribution of hotspots in order to maximize the Raman intensity.

A subsequent approach was to use an additional guide structure patterned on the substrate to aid in depositing the particles on the structure. For instance, Si substrates were developed in patterns of nanorod, nanohump, and photolithographic or nanosphere lithographic microstructures so AgNPs could be deposited in efficient 3D hotspots [8,9,10,11]. Also, poly(oligo(ethylene glycol)methacrylate) (POEGMA) brushes were prepared as substrates for sputtered AgNPs to increase the hotspot density [12], and polystyrene-block-poly(4-vinylpyridine) copolymer (PS-b-P4VP) micelles were also utilized as substrates to form Ag-nanoclusters by the reduction of silver precursors [13]. Moreover, a nanoparticle film was formed on the surface of a non-plasmonic particle assembly, such as iron nanoparticles [14] and reverse micelles of poly(styreneblock-2-vinylpyridine) (PS-b-P2VP) [15]. While these films turned out to be effective in detecting the analyte due to the intensified Raman signal, the methods for fabrication of the SERS substrates were complex and costly.

A cost-effective and functional approach was subsequently proposed as a 3D SERS substrate. The key component of that substrate was Ag-nanospheres (AgNSs) made of single Ag-nanocrystals (AgNCs) via bottom-up assembly by surfactants at an oil–water interface. The resulting colloidal spheres had many hotspots at the gap between each crystal [16,17]. These AgNSs have been employed as the 3D SERS substrate in several different applications, such as the detection of pathogenic bacteria [18], melamine [19], maize toxin [20,21], and drugs in human urine [22].

The limitation of the AgNS approach was that the spheres were dispersible in water, so they were not compatible with samples based on organic solvents. Thus, a technique that can transfer the spheres from the water phase to the organic phase is needed when using SERS to sense organic samples. A number of different techniques have been investigated for the phase transfer of the nanoparticles [23], but only electrostatic interaction-based phase transfer processes relevant to our study have been considered at present.

Depending on the nature of molecules to be transferred in the organic solvent, several different techniques have been introduced. First, Au nanoparticles (AuNPs) modified with negatively charged valine [24] or carboxylate [25] were transferred to the organic solvent, where primary amine was dissolved due to the interaction between the amine and the carboxylate.

Second, the most commonly used molecules in the organic solvent were cationic surfactants, especially tetraoctylammonium bromide (TOAB) and cetrimonium bromide (CTAB). Many previous studies used mercaptocarboxylic acids as capping reagents of AuNPs, so the interaction between cations of the added surfactants in organic solvent and the carboxylate of the particle surface enabled them to be successfully transferred to the organic solvent phase [26,27,28,29,30,31,32]. In addition to mercaptocarboxylic acid, glutathione (GSH) with two carboxylic groups or calixresorcinarene as the macrocycle were employed to cover the exterior of metal-based nanoparticles. In these cases, the nanoparticles protected by those molecules were finally transferred to the organic solvent due to the cation of the added CTAB [33,34].

Similar to the results with cationic surfactants, alkyldimethyl(ferrocenylmethyl) ammonium ions also proved useful for phase transfer of AuNPs capped by thiolated cyclodextrins (CD) [35], and negatively charged polystyrene (PS) microspheres were utilized as carriers for the phase transfer of positively charged hydrophilic particles [36].

However, apparently no research has dealt with the phase transfer of aggregates of nanoparticles rather than single nanoparticles, and the effect of the phase (either aqueous or non-aqueous) in which the added cationic surfactant is dissolved also has not been investigated.

Therefore, the objectives of this study were to make water-dispersible AgNSs, transfer them to an organic solvent via electrostatic interactions, and employ them as a 3D SERS substrate to identify VOCs relevant to plant defense systems.

## 2. Materials and Methods

### 2.1. Material Description

Methyl salicylate standard (CAS Number 119-36-8, 99% purity), Tenax-TA (60/80 mesh, CAS Number 24938-68-9), and chemicals used for manufacturing AgNSs, including sodium oleate (CAS Number 143-19-1), oleic acid (CAS Number 112-80-1, 99% purity), cyclohexane (CAS Number 110-82-7, 99.5% purity), and sodium dodecyl sulfate (CAS Number 151-21-3, 99% purity), were purchased from Sigma Aldrich (St. Louis, MO, USA). Silver nitrate (AgNO3, CAS Number 7761-88-8, 99.7% purity) was obtained from Fisher Scientific (Pittsburgh, PA, USA). All organic solvents, reagents, and chemicals were of analytical grade and used as received without further purification.

### 2.2. Experimental Procedure

#### 2.2.1. Fabrication of AgNSs

The two-step methodology for fabrication of AgNSs was based on previous research [20,21]. To fabricate AgNCs, a mixture, including 0.5 g of AgNO_3_, 10 mL of deionized (DI) water, 0.8 g of sodium oleate, 1 mL of oleic acid, and 5 mL of ethanol, was added to a 20-mL glass vial under agitation. The vial was tightly sealed and stored in an oven at 150 °C overnight. Afterward the vial was washed out with ethanol and fully dried to create a layer of AgNCs at the bottom of the vial. To fabricate AgNSs, 80 mg of AgNCs from the first step was dissolved in 20 mL of cyclohexane, and 560 mg of sodium dodecyl sulfate (SDS) was also dissolved in 100 mL of DI water. These immiscible solutions were mixed and sonicated for 1 h and then heated at 70 °C until the cyclohexane was mostly evaporated. The concentrated AgNSs were finally prepared by repeating the centrifugation and re-dispersion of DI water.

#### 2.2.2. Phase Transfer

The methodology for phase transfer was finally developed based on our previous work [37].

##### Cationic Surfactants in Aqueous Phase

Three cationic surfactants, TOAB, CTAB, and benzalkonium chloride (BKC), were added separately to DI water on a hot plate until they were completely dissolved to prepare a 0.28 M solution of each. Three mixtures were made by mixing 100 µL of AgNSs with 100 µL of each cationic surfactant solution. Each AgNS mixture was vigorously vortexed for 1 min, and then 200 µL of dichloromethane was added into each mixture, and each mixture of immiscible liquids was finally vortexed again for 1 min. The occurrence of phase transfer was confirmed by observing the color change at the bottom from clear to dark. If the AgNSs in DI water were transferred into the dichloromethane, only the transferred part at the bottom was collected and centrifuged to concentrate only the transferred AgNSs, which were finally re-dispersed in 200 µL of dichloromethane for use as a 3D SERS substrate.

##### Cationic Surfactants in Non-Aqueous Phase

The same three cationic surfactants were dissolved in dichloromethane at a level of 0.14 M, and 100 µL of AgNSs were diluted with 100 µL of DI water. Then, 200 µL of each surfactant in dichloromethane solution was mixed with 200 µL of two times diluted AgNS solution, and the mixture was vigorously vortexed for 1 min. Only the transferred AgNS solutions showing significant color change at the bottom were collected by centrifugation, and these were re-dispersed in 200 µL of dichloromethane for use as a 3D SERS substrate.

#### 2.2.3. 3D SERS Substrate

The SERS substrate was based on the complex between transferred AgNSs and Tenax-TA polymer, and three different complex solutions were prepared by dissolving Tenax-TA (1, 2, and 3 mg) into 150 µL of transferred AgNS solution in dichloromethane. A 20-µL volume of each complex solution was drop-casted on a clean quartz substrate and fully dried.

#### 2.2.4. Static Volatile Collection

For the generation of methyl salicylate (MeSA) volatile, 5 µL of the MeSA was dropped inside the jar with 120 mL, and three different SERS substrates were placed inside the jar by facing the substrates to the evaporated reagent for two different collection times (4 h and overnight (approximately 20 h)).

#### 2.2.5. Characterization Analysis

To determine the morphology of the AgNCs and AgNSs before and after phase transfer, carbon-coated 300-mesh grids were treated by glow discharge, and then approximately 2 to 3 µL of the samples (AgNCs, AgNSs, and transferred AgNSs) were applied to the grid. After 10 s, the excess was blotted off with filter paper. The prepared specimens were observed in a JEOL 1200 EX transmission electron microscope (TEM) operated at an acceleration voltage of 100 kV. Electron micrographs were recorded at calibrated magnifications with a 3k slow-scan CCD camera (model 15C, SIA), and particle size was measured with ImageJ software.

To also investigate the surface morphology of the transferred AgNSs, approximately 2 to 3 µL of the samples (transferred AgNSs) were again applied to the new carbon-coated grid. After the drying of the samples on the grid, the specimen was sputter-coated with Pt/Pd 4 nm to improve the image quality and observed in a JEOL JSM-7500F field emission scanning electron microscope (FE-SEM).

To obtain elemental information from the AgNCs, X-ray photoelectron spectroscopy (XPS) analysis of the sample was performed on an Omicron ESCA+ with Mg Ka X-rays at 300 W and with charge neutralization of the sample.

To obtain elemental and crystallographic information from the AgNSs before and after phase transfer, approximately 2 to 3 µL of the samples (AgNSs and transferred AgNSs) were deposited onto TEM grids with a carbon film and dried on a paper filter. The spheres were characterized by FEI TECNAI G2 F20 Super-Twin TEM fitted with a Schottky field emission gun, a 2k × 2k Gatan CCD camera, and an Oxford windowless energy dispersive X-ray spectroscopy (EDX) detector (Oxford X-Max^N^ TSR) with a collection solid angle in the range of 0.3 to 0.7 steradians. The elemental analysis was performed in the area of agglomerated AgNSs and transferred AgNSs at a 200-kV accelerating voltage, and the collected EDX spectra were exported from Oxford AZtec software. In addition to the chemical elements from the TEM-EDX system, electron diffraction patterns were also taken from the AgNSs and transferred AgNSs at a camera length of 200 mm.

#### 2.2.6. UV/Vis Spectroscopy

Both water-dispersible AgNSs and the transferred AgNSs solutions were prepared in a quartz cuvette, and UV/Vis-NIR spectra for both were acquired with a Hitachi U-4100 spectrophotometer.

#### 2.2.7. Raman Spectroscopy

Each substrate after static volatile collection was placed on the stage of a Raman spectroscopy instrument (RamanStation 400 F, Perkin-Elmer, Beaconsfield, Buckinghamshire, UK), which consists of a 256 × 1024 pixel CCD detector and a 175-mW near-infrared (785 nm) laser. All representative spectra that were averaged from seven different spots of the obtained SERS substrate were collected in quadruplicate with 2 s of exposure at a spectral resolution of 4 cm^−1^ in the Raman shift range of 200 to 2000 cm^−1^. All collected spectra were exported from built-in software (The Spectrum v. 6.3, Perkin-Elmer, Beaconsfield, Buckinghamshire, UK) and finally processed with baseline correction and normalization in MATLAB’s bioinformatics toolbox.

## 3. Results and Discussion

### 3.1. Phase Transfer of AgNSs

#### 3.1.1. Cationic Surfactants in Aqueous Solution

**Scheme 1:**
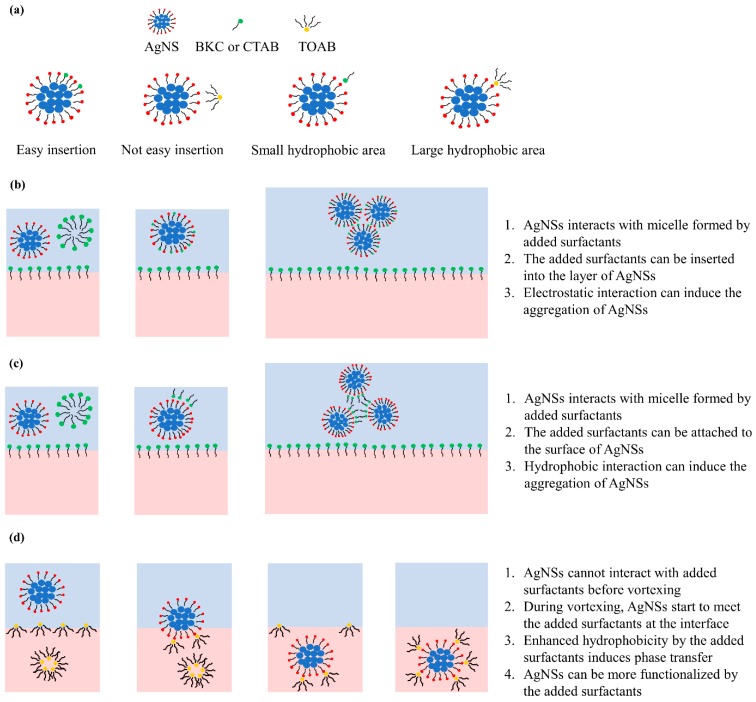
Schemes of several possible interfacial phenomena for phase transfer. (**a**) Effect of the hydrophobic tail area according to the surfactants. (**b**) One possible interaction between AgNS and the added surfactant with a small hydrophobic tail (BKC, CTAB) in the water phase (Blue: water, Pink: dichloromethane): Surfactant insertion into AgNS. (**c**) Another possible interaction between AgNS and the added surfactant with a small hydrophobic tail (BKC, CTAB) in the water phase (Blue: water, Pink: dichloromethane): Surfactant attraction to AgNS. (**d**) Successful phase transfer of AgNS by the added TOAB dissolved in dichloromethane.

**Figure 1:**
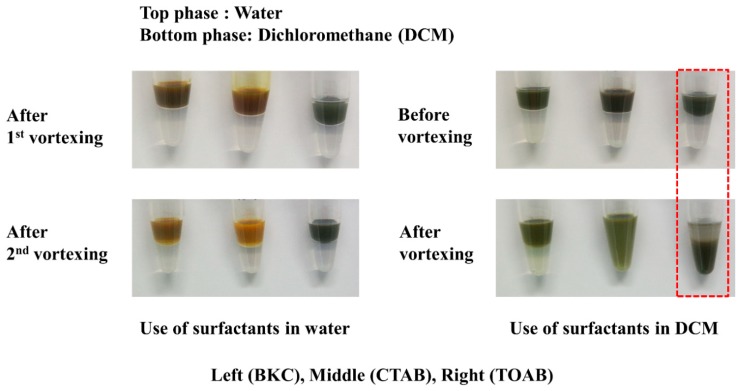
Phase transfer of AgNSs from water to dichloromethane by different cationic surfactants (BKC, CTAB, and TOAB).

Distinct features of phase transfer (Figure 1) depended on the following: (1) What kind of cationic surfactant was used, and (2) in which phase (either aqueous or non-aqueous) the cationic surfactant was dissolved. When the cationic surfactant dissolved in water was added to AgNSs in the water, the phase transfer did not work at all for any of the surfactants. One reason might be low water solubility of each surfactant. In particular, TOAB was not more soluble than the others and required continuous heating to maintain solubility due to its large hydrophobic tail area (Scheme 1a). Results from the other two surfactants were also poor, even though their solubility was somewhat better. The AgNSs may have interacted with the micelle formed by adding surfactant during the first vortexing of the mixtures of AgNSs and added surfactant in water phase (Scheme 1b and c). However, some of the added cationic surfactants may have started to move to the water/dichloromethane interface, and some of them may have interacted with the SDS right after the second vortexing with added dichloromethane. While interacting with the SDS, they may have either been inserted into the spot where the SDS layer had formed or attached to the head of the SDS by electrostatic interaction. The hydrophobic tail area of the other two surfactants was smaller than that of TOAB, so they may have been inserted at the interface where anionic SDS surfactant had already been aligned (Scheme 1a). As a result, the electrostatic interaction between the cationic surfactant of one AgNS and the anionic surfactant of another AgNS may induce aggregation (Scheme 1b). The aggregation of AgNSs may also be explained in a different way (Scheme 1c). The added cationic surfactants may have been attracted to the anionic surfactant by electrostatic interaction, and the hydrophobic tails of the cationic surfactants may have been exposed to the water phase, which is not a favorable situation. Therefore, the modified AgNSs with the added surfactants may have been trying to aggregate together to minimize the exposed hydrophobic area in the water phase. Finally, the aggregates (based on either possible explanation) may have a much greater diameter than the original AgNS, which can be confirmed by a color change from dark green to brown (Figure 1). In accordance with the first explanation, it has been reported that cetyltrimethylammonium chloride (CTACl) cationic surfactant can interact with negatively charged AuNPs, with the aggregation confirmed by a color change from red to blue [38]. Whether the phase transfer can occur during vortexing can be determined largely by how well the AgNSs can be modified with the cationic surfactant to increase hydrophobicity, in which case the aggregates would still have a chance to meet other cationic surfactants aligned at the water/dichloromethane interface. However, there may be no way to make the surfactants cover the surface of the aggregates due to their increased surface area, so inadequate hydrophobicity could be the reason they were not driven to the organic phase.

#### 3.1.2. Cationic Surfactants in Non-Aqueous Solution

**Figure 2:**
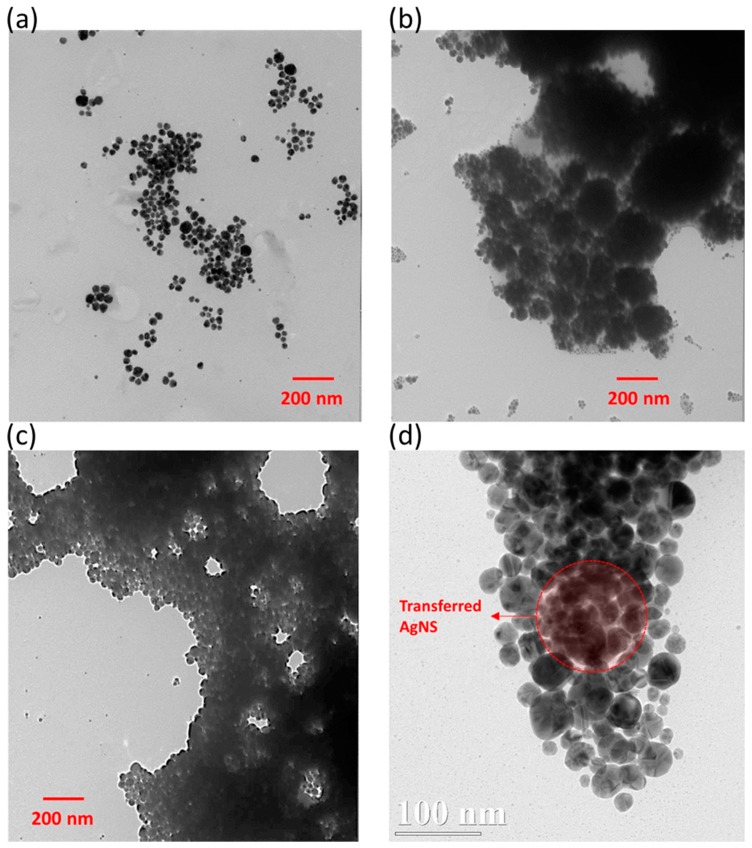
TEM images of AgNCs and AgNSs before/after phase transfer. (**a**): AgNCs, (**b**): AgNSs before phase transfer, (**c**): Transferred AgNSs, (**d**): Transferred AgNSs with high resolution).

A major consideration is that each cationic surfactant had a different effect on the phase transfer (Figure 1). When BKC cationic surfactants were added, phase transfer did not occur at all (no color change). However, the addition of CTAB or TOAB cationic surfactants did result in a color change, and a clear color change was observed in the case of TOAB surfactants, meaning that most of the AgNSs in the water phase were transferred to the organic phase. The key factor in the difference appears to be the hydrophobicity of the surfactants, such that if the AgNSs modified with the cationic surfactant by electrostatic interaction at the water/dichloromethane interface become hydrophobic enough, they are finally moved from the water to the organic phase. Again, the TOAB that was not dissolved in water had four hydrocarbon tails rather than only one tail like the other two surfactants. Thus, if it is assumed that one mole of each cationic surfactant is attached to one mole of the SDS anionic surfactant of the AgNSs, a much greater hydrophobicity can be exerted on the AgNSs in the TOAB case, and a lesser number of moles should be enough to transfer them to the organic phase. The TOAB-induced phase transfer procedure is shown in Scheme 1d. A slight color change was found at the bottom of the organic phase as a result of the addition of CTAB, meaning that only a few AgNSs were transferred to the bottom. This result can be explained similarly, such that a larger number of moles of CTAB might be needed to produce the same hydrophobicity induced by TOAB. The phase transfer induced by TOAB was also confirmed by TEM (Figure 2), and the spherical aggregates representing the AgNSs were found in both cases (before and after phase transfer). Especially, the diameter of the transferred AgNS was estimated based on a high-resolution TEM image (Figure 2d), and the estimated diameter of the transferred AgNS was 124 nm with the averaged diameter of the AgNCs (29 nm). The transferred AgNS was also clearly observed by SEM images, showing spherical aggregates with a diameter of around 100 nm (Supplementary Materials, Figure S2).

**Figure 3:**
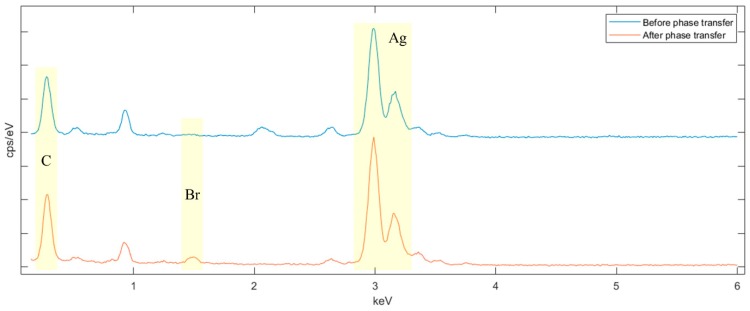
EDX spectra of AgNSs before/after phase transfer.

**Figure 4:**
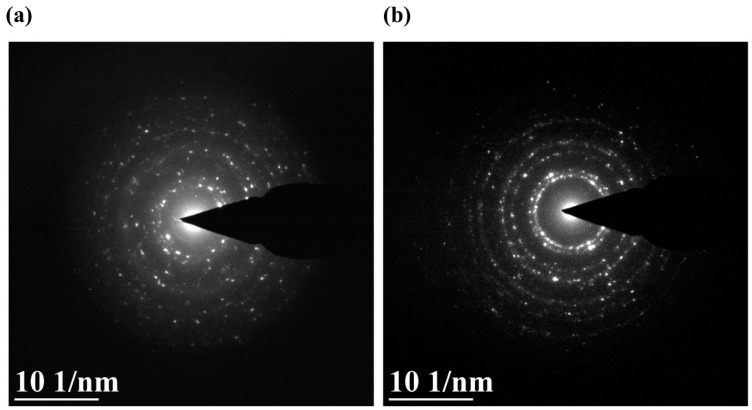
Crystal diffraction pattern of AgNSs (**a**) before phase transfer and (**b**) after phase transfer.

In addition to the several characterization images, elemental analyses from the samples were performed, and the quality of the AgNCs as a main material of the AgNSs was successfully confirmed by unique XPS spectra including several peaks from Ag, C, and O (Supplementary Materials, Figure S3). The successful effect of TOAB was additionally analyzed by EDX (Figure 3), and the bromide peak that was only visible after phase transfer is strong evidence that AgNSs were well-modified with the added TOAB during phase transfer. In addition, the crystallinity of the AgNSs was examined by analyzing the diffraction pattern, and spot and ring patterns were clearly identified from both cases (Figure 4), confirming that the quality of the crystal structure was not affected by the addition of TOAB.

The successful phase transfer of the AgNSs raises the question, how is it possible to transfer AgNSs with diameter above 50 nm by using TOAB? Cheng et al. used TOAB to make AuNPs covered with citrate phase transferred from water to toluene by electrostatic interaction. They found that phase transfer by electrostatic interaction alone was not effective for nanoparticles above 10 nm in diameter due to the decreased surface area to volume ratio [39]. In other words, the TOAB coverage on the surface of the nanoparticles was less for larger particles, contrary to our results. Therefore, the nanoparticles must be considered in detail to elucidate the difference. The AuNPs of Cheng et al. were formed by citrate reduction of HAuCl_4_, so the particles were highly stabilized by the citrate anion uniformly covering them [40]. The citrate anion layer on the AuNPs was also considered to unveil the exact mechanism of formation. The citrate trimer, having two absorbed molecules and one dangling molecule, may be a unit of the layer, with a surface coverage of 2.8 × 10^−6^ mol/m^2^. The central COO^−^ group of the exposed dangling citrate may be the main source for the negative charge [41], and many other polar groups, including hydroxyl and other carboxyl, can be major factors in making the particle hydrophilic. However, the AgNSs used in our experiment were formed by hydrophobic interaction between a cluster of AgNCs and the tail of the SDS anionic surfactants, so how regularly the SDS anionic surfactants were packed at the water–oil interface where the AgNCs were dissolved needs to be known. If the surfactants were sparsely positioned at the interface, a smaller number of the cationic surfactants might have been needed to induce the electrostatic interaction between them, a possibility supported by previous research [42]. The hydrophobic tail of the SDS is not positioned in an orderly way in the oil phase, even in contact with water, so the surface area is about several hundred square angstroms, corresponding to a surface coverage of 3.92 × 10^−7^ mol/m^2^ and comparable to the surface area of citrate with about 10 times difference in quantity. Furthermore, the disorder of the hydrophobic tail inside the oil phase might help the particles transfer to the organic phase due to the more exposed hydrophobic area into the water. This possibility is different from the case of citrate in that some of the polar groups might keep the particles from being hydrophobic even after interacting with the added TOAB. Taken together, these two points may explain how large particles can be transferred from the water to the organic phase.

### 3.2. SERS Application for the Determination of MeSA

**Figure 5:**
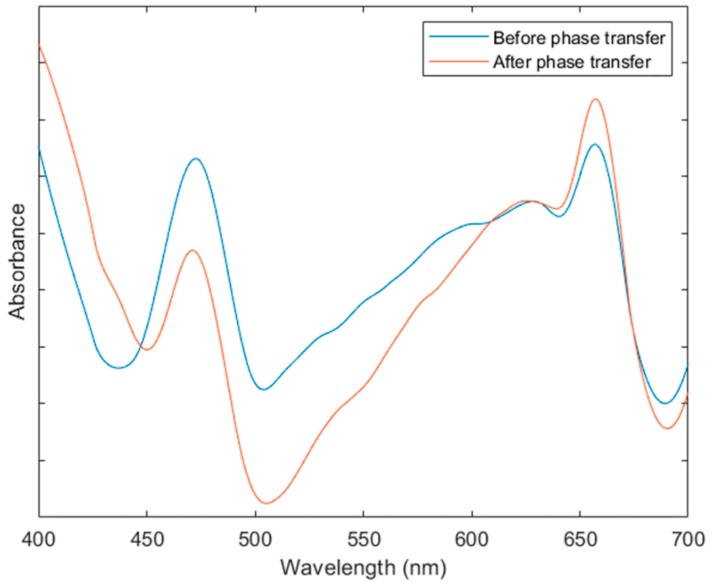
UV/Vis absorption spectrum of AgNSs before/after phase transfer.

To finally utilize the transferred AgNSs as a SERS substrate, the UV/Vis absorption spectra needs to be compared between AgNSs before/after the phase transfer process. The UV/Vis absorption spectra intensity of AgNSs at around 450 nm before phase transfer was higher than after phase transfer (Figure 5). Not all the particles from the water phase could be transferred to the organic phase, so the final concentration after phase transfer was less than the original concentration, resulting in a lower absorption intensity. In addition, the absorption peak around 400 and 500 nm after phase transfer was a little bit red-shifted due to the functionalization of the added surfactants, but the shape of the spectrum was still maintained, similar to the absorption spectra of the AgNSs used as the SERS substrate [18]. We could also observe another peak around 650 nm relevant to particle aggregation [43], so the spherical aggregate of the particles and its aggregation may affect those peaks for both cases. Therefore, the AgNSs after phase transfer were used as a 3D SERS substrate to detect MeSA, an important VOC for plant systematic defense [44]. Tenax TA, poly(2,6-diphenylene oxide), is known to be an effective adsorbent polymer for collecting a wide range of VOCs emitted from plants and foods. It is usually used with GC/MS analysis, and a thermal µ-preconcentrator where the adsorbent is coated has also been developed for the detection of VOCs [45,46]. However, this combined technique requires a thermal desorption step before detecting VOCs, and the configuration is complicated to fabricate. In contrast to GC/MS analysis, our 3D SERS substrate does not need thermal desorption. The 3D SERS substrate was tested to determine whether it can directly measure pre-concentrated MeSA at different concentrations in the adsorbent polymer.

**Figure 6:**
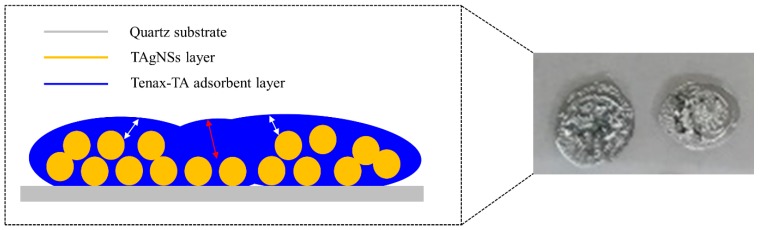
Scheme of transferred AgNSs-polymer complex-based SERS substrate.

**Figure 7:**
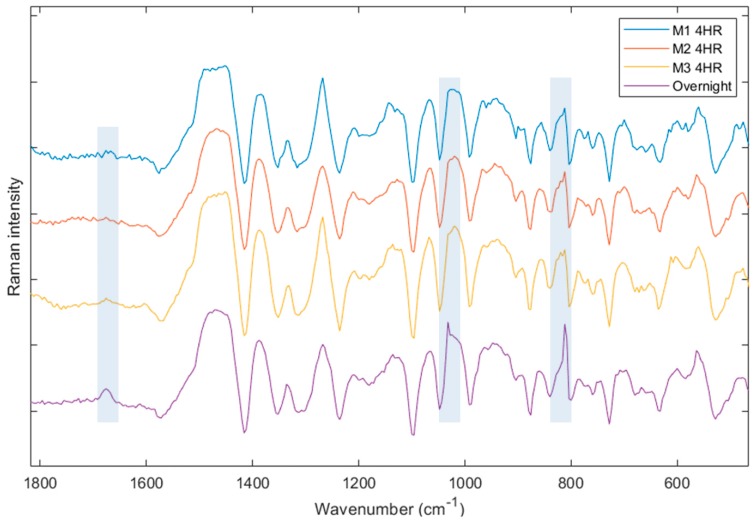
SERS spectra of MeSA VOCs according to the collection times. M1 4HR: Mixture with 1 mg of polymer for 4 h, M2 4HR: Mixture with 2 of mg polymer for 4 h, M3 4HR: Mixture with 3 mg of polymer for 4 h, overnight: Averaged over three concentrations overnight. MeSA standard Raman spectrum is available in the Supplementary Materials, Figure S1.

**Figure 8:**
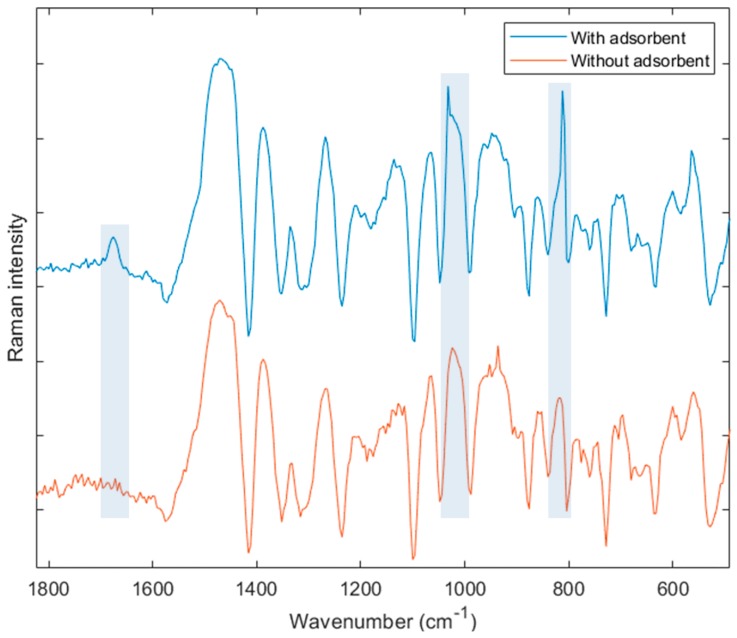
The effect of adsorbent on SERS spectra of MeSA VOC. MeSA standard Raman spectrum is available in the Supplementary Materials, Figure S1.

Due to surface property changes of transferred AgNSs, the added polymer was well-dispersed in the transferred AgNSs solution, and the film from the complex solution was uniformly formed on a quartz substrate. The hydrophobic interaction between phenyl groups of the polymer and hydrophobic tail of the TOAB on transferred AgNSs should be the main force for the formation of the complex, and this notion was confirmed; hydrophobic polymer above a certain concentration or chain length can successfully interact with the hydrophobic core of a lipid membrane similar to the TOAB amphiphilic molecule [47]. The film formed from the complex solution was easily deposited on the quartz substrate immediately after evaporation of the dichloromethane solvent, and the film ultimately deposited on the quartz substrate is shown with the plausible scheme in Figure 6. Transferred AgNSs appeared to be immobilized on the quartz substrate, where they were surrounded by the polymer matrix. This observation is supported by a previous fundamental study about solid film generated from a nanoparticle–polymer complex of the solution, even though the complex was dispersed in aqueous solution, not organic solution like in our case [48]. The solid film was finally tested with MeSA in the vapor phase to determine whether the film could efficiently capture the vapor as it was naturally evaporated from its liquid phase depending on its vapor pressure, and the maximum MeSA VOC can be approximated based on the saturated VOC in a headspace (Supplementary Materials, S1). Four different SERS spectra of the vapor are shown in Figure 7. After 4 h of vapor capture, the case with the highest polymer concentration looked better than the other two cases. The three spectra had similar features, with one unique peak at the 812 cm^−1^ wavenumber region due to out-of-plane deformation of C-H. However, two other peaks at 1032 and 1676 cm^−1^ from in-plane deformation of C-H and stretching of C=O were relatively weak [49]. All three peaks became dominant after overnight collection regardless of the added polymer concentrations, and another small peak was observed at 1620 cm^−1^ from stretching of the phenyl group [49]. Evaporation of a liquid drop of MeSA may take some time to reach a certain vapor pressure, so the amount of volatile produced during 4 h is likely to be lower than that overnight. In addition, another study was done to observe how the use of the adsorbent polymer could affect the enhancement of the SERS spectra, and the comparison of whether or not to use the adsorbent is shown in Figure 8. If only transferred AgNSs were used as a SERS substrate, no significant peaks from the MeSA VOC were not identified even for overnight collection. However, the addition of the adsorbent layer might be able to improve the VOC collection efficiency, producing three significant MeSA VOC peaks from the overnight collection. An analytical enhancement factor (AEF) by the SERS substrate can also be approximated from the saturated VOC concentration in a headspace, finally providing 7.53 × 10^6^ AEF (Supplementary Materials, S2). However, an assumption that the MeSA VOC can be absorbed on the surface of the transferred AgNSs with a sub-monolayer should be addressed prior to the AEF calculation [50].

Nevertheless, the uniformity of the distance between the polymer surface and the transferred AgNSs surface may factor into the difference in spectral intensity. If the adsorbed molecule at the surface of the polymer is located far away from the transferred AgNSs (Figure 6, red double arrow), the SERS intensity from the molecule may be weaker than from a molecule near the transferred AgNSs (Figure 6, white double arrow) due to decreased light penetration [51,52]. Thus, uniformity should be improved to intensify the SERS signal, and SERS mapping can be an alternative option to prove the homogeneity of the SERS substrate. Nevertheless, the detection of MeSA in the vapor phase by using simple complexes between transferred AgNSs and adsorbent is notable, and multiplex detection of VOCs warrants further study.

## 4. Conclusions

Spherical aggregates of single nanoparticles were successfully transferred from aqueous phase to a non-aqueous phase by electrostatic interaction induced by TOAB surfactants. The key idea is to use an aggregate covered by surfactants with an enlarged surface area, a useful concept for the phase transfer of large nanoparticles in many applications. The transferred AgNSs were well-mixed with polymer adsorbent, and the complex between the AgNSs and polymer adsorbent was successfully used as an ADS-SERS substrate. After static collection of the volatiles of MeSA by the substrate, the spectra at several wavenumbers could clearly identify the MeSA, and the peak intensity at each wavenumber was dramatically increased for overnight collection, suggesting the possibility for quantitative detection of MeSA volatiles. The ADS-SERS technique shows promise for a sensor to detect MeSA volatile, and other VOCs in food and agriculture should be tested in the near future.

## Supplementary Materials

The following are available online at https://www.mdpi.com/2079-4991/9/11/1621/s1, Figure S1: MeSA Raman spectra from KnowItAll software, Figure S2: SEM images of the transferred AgNSs, Figure S3: XPS spectra of the AgNCs.

## References

[1] Mosier-Boss P.A. (2017). Review of SERS substrates for chemical sensing. Nanomaterials.

[2] Chou A., Jaatinen E., Buividas R., Seniutinas G., Juodkazis S., Izake E.L., Fredericks P.M. (2012). SERS substrate for detection of explosives. Nanoscale.

[3] Lee H.K., Lee Y.H., Koh C.S.L., Phan-Quang G.C., Han X., Lay C.L., Sim H.Y.F., Kao Y.-C., An Q., Ling X.Y. (2019). Designing surface-enhanced Raman scattering (SERS) platforms beyond hotspot engineering: Emerging opportunities in analyte manipulations and hybrid materials. Chem. Soc. Rev..

[4] Kuttner C. (2018). Plasmonics in Sensing: From Colorimetry to SERS Analytics. Plasmonics.

[5] Xu H., Aizpurua J., Käll M., Apell P. (2000). Electromagnetic contributions to single-molecule sensitivity in surface-enhanced Raman scattering. Phys. Rev. E.

[6] Jiang X., Lai Y., Yang M., Yang H., Jiang W., Zhan J. (2012). Silver nanoparticle aggregates on copper foil for reliable quantitative SERS analysis of polycyclic aromatic hydrocarbons with a portable Raman spectrometer. Analyst.

[7] Chen J., Qin G., Wang J., Yu J., Shen B., Li S., Ren Y., Zuo L., Shen W., Das B. (2013). One-step fabrication of sub-10-nm plasmonic nanogaps for reliable SERS sensing of microorganisms. Biosens. Bioelectron..

[8] Tang H., Meng G., Huang Q., Zhang Z., Huang Z., Zhu C. (2012). Arrays of Cone-Shaped ZnO Nanorods Decorated with Ag Nanoparticles as 3D Surface-Enhanced Raman Scattering Substrates for Rapid Detection of Trace Polychlorinated Biphenyls. Adv. Funct. Mater..

[9] Li Z., Meng G., Huang Q., Hu X., He X., Tang H., Wang Z., Li F. (2015). Ag Nanoparticle-Grafted PAN-Nanohump Array Films with 3D High-Density Hot Spots as Flexible and Reliable SERS Substrates. Small.

[10] Zhang Q., Lee Y.H., Phang I.Y., Lee C.K., Ling X.Y. (2014). Hierarchical 3D SERS Substrates Fabricated by Integrating Photolithographic Microstructures and Self-Assembly of Silver Nanoparticles. Small.

[11] Bechelany M., Brodard P., Philippe L., Michler J. (2009). Extended domains of organized nanorings of silver grains as surface-enhanced Raman scattering sensors for molecular detection. Nanotechnology.

[12] Zhang Q., Wang X.-D., Tian T., Chu L.-Q. (2017). Incorporation of multilayered silver nanoparticles into polymer brushes as 3-dimensional SERS substrates and their application for bacteria detection. Appl. Surf. Sci..

[13] Cho W.J., Kim Y., Kim J.K. (2011). Ultrahigh-density array of silver nanoclusters for SERS substrate with high sensitivity and excellent reproducibility. ACS Nano.

[14] Fan Z., Senapati D., Khan S.A., Singh A.K., Hamme A., Yust B., Sardar D., Ray P.C. (2013). Popcorn-Shaped Magnetic Core–Plasmonic Shell Multifunctional Nanoparticles for the Targeted Magnetic Separation and Enrichment, Label-Free SERS Imaging, and Photothermal Destruction of Multidrug-Resistant Bacteria. Chem. A Eur. J..

[15] Yap F.L., Thoniyot P., Krishnan S., Krishnamoorthy S. (2012). Nanoparticle cluster arrays for high-performance SERS through directed self-assembly on flat substrates and on optical fibers. Acs Nano.

[16] Wang X., Zhuang J., Peng Q., Li Y. (2005). A general strategy for nanocrystal synthesis. Nature.

[17] Bai F., Wang D., Huo Z., Chen W., Liu L., Liang X., Chen C., Wang X., Peng Q., Li Y. (2007). A Versatile Bottom-up Assembly Approach to Colloidal Spheres from Nanocrystals. Angew. Chem. Int. Ed..

[18] Wang Y., Lee K., Irudayaraj J. (2010). Silver nanosphere SERS probes for sensitive identification of pathogens. J. Phys. Chem. C.

[19] Liu Y., Zhang Y., Ding H., Xu S., Li M., Kong F., Luo Y., Li G. (2013). Self-assembly of noble metallic spherical aggregates from monodisperse nanoparticles: Their synthesis and pronounced SERS and catalytic properties. J. Mater. Chem. A.

[20] Lee K.-M., Herrman T.J., Bisrat Y., Murray S.C. (2014). Feasibility of surface-enhanced raman spectroscopy for rapid detection of aflatoxins in maize. J. Agric. Food Chem..

[21] Lee K.-M., Herrman T.J. (2016). Determination and prediction of fumonisin contamination in maize by surface–enhanced Raman spectroscopy (SERS). Food Bioprocess Technol..

[22] Han Z., Liu H., Wang B., Weng S., Yang L., Liu J. (2015). Three-dimensional surface-enhanced Raman scattering hotspots in spherical colloidal superstructure for identification and detection of drugs in human urine. Anal. Chem..

[23] Yang J., Lee J.Y., Ying J.Y. (2011). Phase transfer and its applications in nanotechnology. Chem. Soc. Rev..

[24] Kumar A., Mukherjee P., Guha A., Adyantaya S., Mandale A., Kumar R., Sastry M. (2000). Amphoterization of colloidal gold particles by capping with valine molecules and their phase transfer from water to toluene by electrostatic coordination with fatty amine molecules. Langmuir.

[25] Mayya K.S., Caruso F. (2003). Phase transfer of surface-modified gold nanoparticles by hydrophobization with alkylamines. Langmuir.

[26] Yao H., Momozawa O., Hamatani T., Kimura K. (2000). Phase transfer of gold nanoparticles across a water/oil interface by stoichiometric ion-pair formation on particle surfaces. Bull. Chem. Soc. Jpn..

[27] Yao H., Momozawa O., Hamatani T., Kimura K. (2001). Stepwise size-selective extraction of carboxylate-modified gold nanoparticles from an aqueous suspension into toluene with tetraoctylammonium cations. Chem. Mater..

[28] Devarajan S., Vimalan B., Sampath S. (2004). Phase transfer of Au–Ag alloy nanoparticles from aqueous medium to an organic solvent: Effect of aging of surfactant on the formation of Ag-rich alloy compositions. J. Colloid Interface Sci..

[29] Cheng H.-W., Schadt M.J., Young K., Luo J., Zhong C.-J. (2015). Determination of ion pairing on capping structures of gold nanoparticles by phase extraction. Analyst.

[30] Cheng H.-W., Schadt M.J., Zhong C.-J. (2015). Titration of gold nanoparticles in phase extraction. Analyst.

[31] Guo H., Xing B., White J.C., Mukherjee A., He L. (2016). Ultra-sensitive determination of silver nanoparticles by surface-enhanced Raman spectroscopy (SERS) after hydrophobization-mediated extraction. Analyst.

[32] Yao Q., Yuan X., Yu Y., Yu Y., Xie J., Lee J.Y. (2015). Introducing amphiphilicity to noble metal nanoclusters via phase-transfer driven ion-pairing reaction. J. Am. Chem. Soc..

[33] Yuan X., Luo Z., Zhang Q., Zhang X., Zheng Y., Lee J.Y., Xie J. (2011). Synthesis of highly fluorescent metal (Ag, Au, Pt, and Cu) nanoclusters by electrostatically induced reversible phase transfer. ACS Nano.

[34] Ermakova A.M., Morozova J.E., Shalaeva Y.V., Syakaev V.V., Nizameev I.R., Kadirov M.K., Antipin I.S., Konovalov A.I. (2017). The supramolecular approach to the phase transfer of carboxylic calixresorcinarene-capped silver nanoparticles. Colloids Surf. A Physicochem. Eng. Asp..

[35] Liu J., Alvarez J., Ong W., Román E., Kaifer A.E. (2001). Phase transfer of hydrophilic, cyclodextrin-modified gold nanoparticles to chloroform solutions. J. Am. Chem. Soc..

[36] Sapoletova N.A., Kushnir S.E., Kushnir A.E., Kocherginskaya P.B., Kazin P.E., Napolskii K.S. (2016). Simple phase transfer of nanoparticles from aqueous to organic media using polymer colloids as carriers. RSC Adv..

[37] Park J., Thomasson J.A., Lee K.-M. Volatile Detection from Plant Headspace with Modified Surface-Enhanced Raman Spectroscopy. Proceedings of the 2017 ASABE Annual International Meeting.

[38] Kazakova J., García-Povea A., Fernández-Palacios M., Villar-Navarro M., Carnerero J.M., Jimenez-Ruiz A., Prado-Gotor R. (2017). A colorimetric study of the interaction of cationic and anionic surfactants with anionic gold nanoparticles. Colloid Polym. Sci..

[39] Cheng W., Wang E. (2004). Size-dependent phase transfer of gold nanoparticles from water into toluene by tetraoctylammonium cations: A wholly electrostatic interaction. J. Phys. Chem. B.

[40] Frens G. (1973). Controlled nucleation for the regulation of the particle size in monodisperse gold suspensions. Nat. Phys. Sci..

[41] Park J.-W., Shumaker-Parry J.S. (2014). Structural study of citrate layers on gold nanoparticles: Role of intermolecular interactions in stabilizing nanoparticles. J. Am. Chem. Soc..

[42] De Aguiar H.B., Strader M.L., de Beer A.G., Roke S. (2011). Surface structure of sodium dodecyl sulfate surfactant and oil at the oil-in-water droplet liquid/liquid interface: A manifestation of a nonequilibrium surface state. J. Phys. Chem. B.

[43] Navarro J.R., Werts M.H. (2013). Resonant light scattering spectroscopy of gold, silver and gold–silver alloy nanoparticles and optical detection in microfluidic channels. Analyst.

[44] Park S.-W., Kaimoyo E., Kumar D., Mosher S., Klessig D.F. (2007). Methyl salicylate is a critical mobile signal for plant systemic acquired resistance. Science.

[45] Alfeeli B., Jain V., Johnson R.K., Beyer F.L., Heflin J.R., Agah M. (2011). Characterization of poly (2, 6-diphenyl-p-phenylene oxide) films as adsorbent for microfabricated preconcentrators. Microchem. J..

[46] Chae M.-S., Kim J., Yoo Y.K., Kang J.Y., Lee J.H., Hwang K.S. (2015). A micro-preconcentrator combined olfactory sensing system with a micromechanical cantilever sensor for detecting 2, 4-dinitrotoluene gas vapor. Sensors.

[47] Drenscko M., Loverde S.M. (2019). Molecular dynamics simulations of the interaction of phospholipid bilayers with polycaprolactone. Mol. Simul..

[48] Berret J.-F., Yokota K., Morvan M., Schweins R. (2006). Polymer− nanoparticle complexes: From dilute solution to solid state. J. Phys. Chem. B.

[49] Varghese H.T., Panicker C.Y., Philip D., Mannekutla J.R., Inamdar S. (2007). IR, Raman and SERS studies of methyl salicylate. Spectrochim. Acta A Mol. Biomol. Spectrosc..

[50] Kuttner C., Höller R.P., Quintanilla M., Schnepf M.J., Dulle M., Fery A., Liz-Marzán L.M. (2019). SERS and plasmonic heating efficiency from anisotropic core/satellite superstructures. Nanoscale.

[51] Xia D., Guo Q.H., Ge M., Yuan Y.X., Xu M.M., Yao J.L. (2016). On-line sensitive detection of aromatic vapor through PDMS/C3H7S-assisted SERS amplification. RSC Adv..

[52] Li M., Qiu Y., Fan C., Cui K., Zhang Y., Xiao Z. (2018). Design of SERS nanoprobes for Raman imaging: Materials, critical factors and architectures. Acta Pharm. Sin. B.

