# Supplementary material for: Complexes Formed by Hydrophobic Interaction between Ag-Nanospheres and Adsorbents for the Detection of Methyl Salicylate VOC
Source: Nanomaterials (Basel). 2019 Nov 15;9(11):1621. doi: 10.3390/nano9111621 (PMC6915383; doi:10.3390/nano9111621)
Supplement: Supplementary file 1 [file nanomaterials-09-01621-s001.pdf]

nanomaterials

MDPI

Article

Complexes Formed by Hydrophobic Interaction between Ag-Nanospheres and Adsorbents for the Detection of Methyl Salicylate VOC

Jinhyuk Park [1,*], J. Alex Thomasson [1], Sandun Fernando [1], Kyung-Min Lee [2] and Timothy J. Herrman [2]

[1] Department of Biological and Agricultural Engineering, Texas A&M University, College Station, TX 77843, USA; thomasson@tamu.edu (J.A.T.); sfernando@tamu.edu (S.F.)

[2] Office of the Texas State Chemist, Texas A&M AgriLife Research, Texas A&M University System, College Station, Texas 77841, USA; kml@otsc.tamu.edu (K.L.); tjh@otsc.tamu.edu (T.J.H.)

[*] Correspondence: jjinbba202@tamu.edu; Tel.: +1-979-224-7055 (J.P.)

Supplementary Materials

S1 MeSA VOC concentration approximation

Maximum limit based on the assumption that the vapor was generated due to its own vapor pressure

$$\therefore\ C_{VOC,max}[ppmv] = \frac{P_{VOC}}{P_{atm}} \times 10^6$$

$$V_{VOC} = volume\ of\ VOC\ [L], \qquad V_{Total} = total\ volume\ of\ the\ chamber = 120 \times 10^{-3}[L]$$

$$P_{atm} = standard\ atmosphere = 1\ [atm],\ \ P_{VOC} = vapor\ pressure\ of\ VOC\ [atm],$$

$$\mathrm{R = Ideal\ gas\ constant} = 0.0821\left[\frac{L\ atm}{K\ mole}\right],\ \ \mathrm{T = Absolute\ temperature} = 298\ [\mathrm{K}]\ \mathrm{at}\ 25\ °\mathrm{C}$$

$$\therefore\ MeSA_{VOC,max}[ppmv] = 0.000045\ \mathrm{atm} = 45\ \mathrm{ppmv}$$

S2 Analytical enhancement factor (AEF) calculation

Based on the previous literature, AEF for saturated VOC can be expressed as follows [1].

$$AEF = \left[\frac{I_{SERS}}{I_{Raman}}\right] \times \left[\frac{C_{Raman}}{C_{SERS}}\right]$$

where C = concentration, I = Raman signal intensity

To calculate C_{SERS}, one assumption should be addressed such that the maximum concentration of the MeSA VOC can be produced from a liquid drop of the MeSA after overnight, and the produced VOC can finally interact with the SERS substrate, inducing the SERS signal. Therefore, the concentration is based on the theoretical calculation by the above equation.

$$P_{atm} = standard\ atmosphere = 1\ [atm],\ \ P_{VOC} = vapor\ pressure\ of\ VOC\ [atm],$$

$$\mathrm{R = Ideal\ gas\ constant} = 0.0821\left[\frac{L\ atm}{K\ mole}\right],\ \ \mathrm{T = Absolute\ temperature} = 298\ [\mathrm{K}]\ \mathrm{at}\ 25\ °\mathrm{C}$$

nanomaterials

MDPI

$$C_{SERS} = \frac{P_{VOC}}{RT} = 1.84 \times 10^{-6}\ mol/L$$

To calculate C_{Raman}, same volume of the MeSA liquid drop that was used as the VOC source in SERS experiment was again used to measure standard Raman signal, so below equation needs to be used.

$$C_{Raman} = \frac{Density\ of\ MeSA}{Molecular\ weight\ of\ MeSA} = \frac{1.174}{152} = 7.7 \times 10^{-3}\ mol/ml$$

Therefore, AEF can be finally calculated as below.

$$AEF = [1.8] \times \left[\frac{7.7}{1.84 \times 10^{-6}}\right] = 7.53 \times 10^{6}$$

S3 MeSA Raman spectra

The spectra of MeSA can be imported from commercial Raman libraries (KnowItAll Informatics System 2018, Bio-Rad Laboratories, Inc., USA). As shown in Figure S1, three dominant peaks are located at the wavenumber of 1676, 1033 and 810 cm^{-1}.

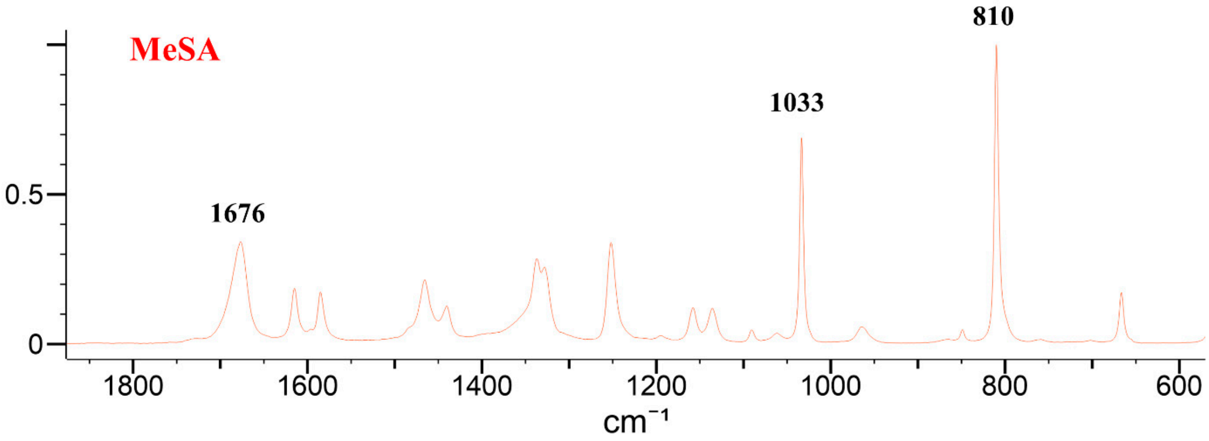

Figure S1. MeSA Raman spectra from KnowItAll software

S4 SEM images of the transferred AgNSs

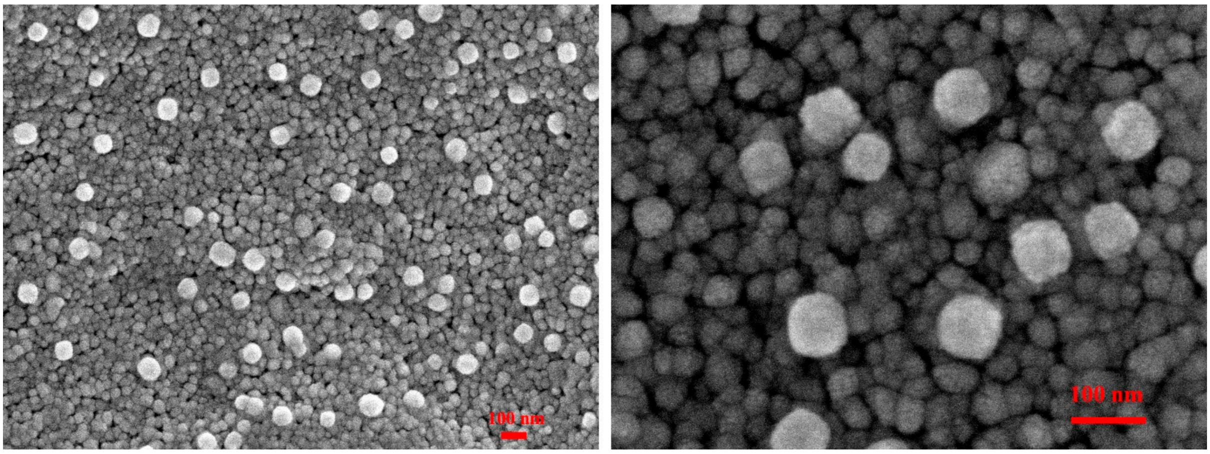

nanomaterials

MDPI

Figure S2. SEM images of the transferred AgNSs

S5 XPS images of the AgNCs

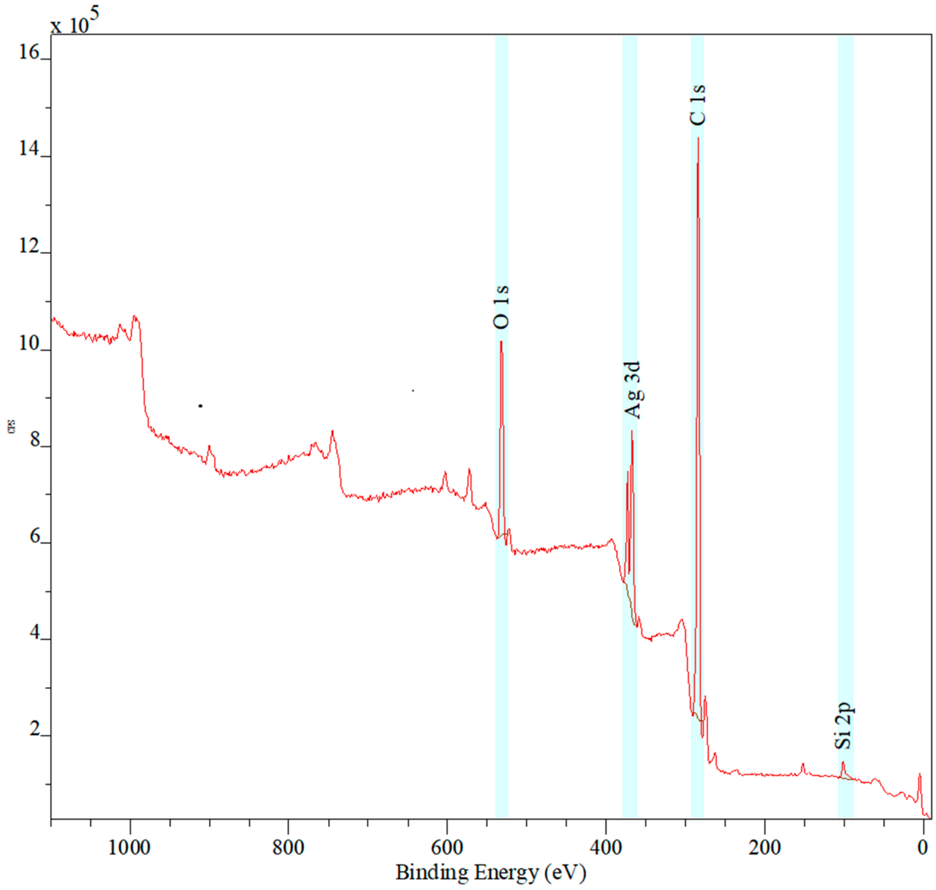

Figure S3. XPS spectra of the AgNCs

Reference

1. Koh, C.S.L.; Lee, H.K.; Han, X.; Sim, H.Y.F.; Ling, X.Y. Plasmonic nose: integrating the MOF-enabled molecular preconcentration effect with a plasmonic array for recognition of molecular-level volatile organic compounds. *Chemical Communications* **2018**, *54*, 2546-2549.
